# BABAR: an R package to simplify the normalisation of common reference design microarray-based transcriptomic datasets

**DOI:** 10.1186/1471-2105-11-73

**Published:** 2010-02-03

**Authors:** Mark J Alston, John Seers, Jay CD Hinton, Sacha Lucchini

**Affiliations:** 1 Foodborne Bacterial Pathogens, Institute of Food Research, Norwich Research Park, Norwich, NR4 7UA, UK; 2 Bioinformatics & Statistics, Institute of Food Research, Norwich Research Park, Norwich, NR4 7UA, UK; 3 Integrated Biology of the GI Tract, Institute of Food Research, Norwich Research Park, Norwich, NR4 7UA, UK; 4 Current address: The Genome Analysis Centre, Norwich Research Park, Norwich, NR4 7UH, UK; 5 Current address: Department of Microbiology, Moyne Institute of Preventive Medicine, School of Genetics and Microbiology, Trinity College, Dublin 2, Ireland

## Abstract

**Background:**

The development of DNA microarrays has facilitated the generation of hundreds of thousands of transcriptomic datasets. The use of a common reference microarray design allows existing transcriptomic data to be readily compared and re-analysed in the light of new data, and the combination of this design with large datasets is ideal for 'systems'-level analyses. One issue is that these datasets are typically collected over many years and may be heterogeneous in nature, containing different microarray file formats and gene array layouts, dye-swaps, and showing varying scales of log_2_- ratios of expression between microarrays. Excellent software exists for the normalisation and analysis of microarray data but many data have yet to be analysed as existing methods struggle with heterogeneous datasets; options include normalising microarrays on an individual or experimental group basis. Our solution was to develop the Batch Anti-Banana Algorithm in R (*BABAR*) algorithm and software package which uses cyclic loess to normalise across the complete dataset. We have already used *BABAR *to analyse the function of *Salmonella *genes involved in the process of infection of mammalian cells.

**Results:**

The only input required by *BABAR *is unprocessed GenePix or BlueFuse microarray data files. *BABAR *provides a combination of 'within' and 'between' microarray normalisation steps and diagnostic boxplots. When applied to a real heterogeneous dataset, *BABAR *normalised the dataset to produce a comparable scaling between the microarrays, with the microarray data in excellent agreement with RT-PCR analysis. When applied to a real non-heterogeneous dataset and a simulated dataset, *BABAR*'s performance in identifying differentially expressed genes showed some benefits over standard techniques.

**Conclusions:**

*BABAR *is an easy-to-use software tool, simplifying the simultaneous normalisation of heterogeneous two-colour common reference design cDNA microarray-based transcriptomic datasets. We show *BABAR *transforms real and simulated datasets to allow for the correct interpretation of these data, and is the ideal tool to facilitate the identification of differentially expressed genes or network inference analysis from transcriptomic datasets.

## Background

DNA microarrays enable the simultaneous measurement of expression levels for many thousands of genes at a given instant in any biological system, revealing the expression state of those features as determined by statistical significance inference [1-3]. By taking many such snapshots over a number of experimental conditions and applying the appropriate analyses (see [4] for an overview), groups of genes with similar expression profiles can be detected to suggest the function or regulation of uncharacterised genes. Analysis of such transcriptomic datasets, or compendia [5,6], allows the expression patterns for particular genes to be compared, offering an ideal way to reveal the higher levels of organisation in an organism by a 'systems'-level approach.

Dual experimental approaches have been used for two-colour transcriptomic microarrays: one makes a direct comparison between complementary DNA (cDNA) samples (two-channel experiments), and the other uses a common reference in one channel of every microarray (one-channel experiments) [7]. *BABAR *was developed to improve the value of data generated by the common reference design which uses the same genomic DNA (gDNA), or pooled RNA, hybridized to every microarray [8]. The power of this design is that it enables the easy comparison between, or within, microarray datasets [9]. The investigator can assemble any combination of data derived from microarrays, making it possible to test new hypotheses. The common reference design is also ideal for 'systems'-level analyses. For example, the context likelihood of relatedness (CLR) algorithm infers prokaryotic transcriptional regulatory networks from this type of compendium of gene expression profiles, or other one-channel designs such as Affymetrix arrays, collected under various experimental conditions [10].

For analytical purposes, we consider common reference experiments to be of a pseudo-direct design such that if the intensity values for a pair of common reference microarrays are plotted on a graph of condition 1 (Red_1_/Green_1_; R_1_/G_1_) versus condition 2 (R_2_/G_2_), where G_x _is the reference channel, then non-differentially expressed genes should map onto a line of slope (R_1_/G_1_)/(R_2_/G_2_) ~ 1. However, experimental data are usually more complex: the data points can diverge from the ideal line in an intensity-dependent manner resulting in a 'banana-shaped' distribution with a non-zero intercept, with non-differentially expressed genes showing considerable scattering around this line and the scatter patterns varying between microarray slides. The intensity relationship between the microarrays is best seen in an MA-plot [11], which is a scatterplot of the intensity log-ratio M = log_2 _{(R_1_/G_1_)/(R_2_/G_2_)} versus the mean log intensity A = log_2 _√{(R_1_/G_1_) × (R_2_/G_2_)}. The focus of the MA-plot is on the symmetrical distribution about the line where M = 0 (i.e. equal expression), but such a plot for a pair of common reference microarrays can show little symmetry due to intensity and spatial variation. Such systematic bias arises from several factors: background effects on the microarray, experimental variation, sample preparation and the fluorescent labelling and printing processes. One example of experimental variation that is of particular relevance to this study involves microarrays that utilize RNA isolated from a challenging environment, such as from bacteria within a mammalian cell. The amount of RNA extracted in such cases is typically small (< 5 μg) [12,13] and the resulting labelled cDNA can give rise to considerable variation between replicate spots on different microarrays. This phenomenon manifests itself as low intensity signals, a high background, or both, and results in a reduction of the distribution of log-ratios of expression. A likely reason for the aberrant labelling is the presence of contaminants associated with the RNA sample (e.g. DNA, carbohydrates or proteins) that can inhibit enzymatic reactions and lead to poor Cy-dye incorporation [14].

Normalisation is the process of removing systematic bias from microarray data. This is achieved by transforming the data values as prescribed by a given algorithm applied 'within' microarrays (applied to each microarray) or 'between' microarrays. Two-colour microarrays are often normalised by applying Locally Estimated Scatterplot Smoothing (loess) to the MA-plot for an entire microarray (global loess) or to the MA-plot for each print-tip group of DNA elements upon a given microarray (print-tip loess). In both cases the algorithm fits a line to the local neighbourhood of each data point and combines these lines to generate a loess curve that follows the central trend across the data. Given the assumption that the majority of genes do not respond to a given experimental condition and should therefore be centred about M = 0, the loess curve is used to adjust the values for each feature accordingly. However, the loess normalisation assumes that the M-value is independent of the A-value. This is not true for common reference microarrays because while the signal for the cDNA channel can vary greatly for each feature, the signal for the gDNA reference channel is more or less constantly high. This results in small M-values corresponding to small A-values, and large M-values corresponding to large A-values; hence the loess cannot be applied to *individual *common reference microarrays.

Therefore, a useful modification of the loess is the cyclic loess normalisation which performs the loess on each unique pair of microarrays in a dataset in order to form the normalising relation [15]. It has been applied to Affymetrix datasets but in principle is equally applicable to pairs of spotted cDNA microarray datasets [15,16]. Such a 'complete data method' [15] appears ideal for simultaneously normalising data across large, heterogeneous common reference datasets, where individual microarrays can show very different spreads and distributions of M-values.

However, consider the example of a pair of microarrays (one a 'control', the other a 'treatment') each with a median M-value close to zero but with very different spreads in their respective M-values. Is this due to technical or biological variation? If the same gene was the most highly expressed feature on both microarrays then without normalisation it would be incorrectly interpreted as being differentially expressed, but imparting identical distributional measures to these microarrays may remove the biological variation in which we are interested.

The method of normalisation must be carefully chosen and a number of 'between arrays' normalisation methods are available. Such methods include scale-normalisation which scales the M-values so that each microarray has the same median absolute deviation, quantile-normalisation which makes the distribution of M-values for each microarray identical, and cyclic loess normalisation (discussed above) which is less aggressive in its normalisation.

Excellent free software exists for the normalisation and analysis of microarray data; for example, Bioconductor [17] is a collection of R [18] packages and includes the *limma *package (Linear Models for Microarray Analysis [19]) which implements global loess, print-tip loess, quantile and scale normalisation. However, working with multiple gene microarray layouts in *limma *is very difficult; for example, given a number of GenePix files described by just two gene array layout (.gal) files, each .gal file and its associated microarray results files must be input and normalised separately. Then prior to merging the two resulting MAList objects, the datasets must be aligned by gene ID, must be of exactly the same length and should contain the same gene IDs.

There is a real need for an application that can automatically (i.e. easily) handle heterogeneous datasets in a robust way. *BABAR *implements the cyclic loess algorithm, normalising each unique pair of microarrays across the whole dataset. We developed *BABAR *to simplify the normalisation process for such datasets by providing a combination of 'within' and 'between' microarray normalisation steps, allowing scientists without a background in statistical programming to focus on the interpretation of results.

## Implementation

### Running *BABAR*

*BABAR *is a software package implemented in R and is available as a zip file (see Additional file 1). Its only dependency is on the *limma *package. As well as the documentation provided with the package, full instructions and an example of running *BABAR *are given in Additional file 2.

### Input and handling of microarray data files

*BABAR *simply requires the unprocessed microarray data files generated by GenePix (Molecular Devices, CA, USA) and/or BlueFuse (BlueGnome Limited, Cambridge, UK) microarray image analysis software as its input. For GenePix files, only the median background and median foreground columns are read for both channels, along with the corresponding Block, Column and Row data. For BlueFuse files, the AMPCH1 and AMPCH2 columns are read along with the corresponding BLOCK, SUBGRIDCOL and SUBGRIDROW data. In this way, the use of gene array layout files is avoided and *BABAR *is able to handle any combination of GenePix and BlueFuse files. To ensure that *mixtures *of GenePix and BlueFuse files were handled correctly, a test-set of microarrays was scanned independently by both imaging software packages. Combinations of GenePix and BlueFuse-derived microarray data were run through *BABAR *and the results compared. No significant differences were seen between GenePix-only, Bluefuse-only, and datasets that were mixtures of these formats; the few small discrepancies simply reflected the different spot-finding software algorithms used by GenePix and BlueFuse and not the *BABAR *normalisation itself (results not shown).

### The *BABAR *algorithm

Here we describe the algorithm and the rationale for normalisation via a number of steps carried out 'within' microarrays (steps 1-5) and 'between' microarrays (steps 6-8):

#### 1 - Background correction

(GenePix only) Intensity values for each gene are background-corrected by subtraction of the median background from the median foreground for both channels. In addition, any feature where the background lies within a user-defined number of standard deviations of the foreground is 'flagged' by *BABAR *(see below). The BlueFuse scanning software automatically carries out background correction via Bayesian statistical methods.

#### 2 - Removal of 'flagged' features

Features are excluded from the normalisation process if: (GenePix only) they are identified in 'step 1' or were flagged by the software as either -50, -75 or -100 ('not found', 'absent' or 'bad', respectively); (Genepix and BlueFuse) features have names like 'EMPTY', 'empty', 'ctrl-' and 'Ctrl-'; (BlueFuse only) they are flagged as 'E' (the lowest spot quality measure).

#### 3 - Automatic reference channel detection (optional)

The same reference sample is used across our datasets. In our hands, for any given microarray, the reference (gDNA) channel always has a higher median value compared to the signal channel. This enables the log_2_-ratios to be automatically calculated in a consistent fashion, even when dye-swaps are present by accident or design.

#### 4 - Block-by-block median centering

Log_2_-ratios (signal channel/gDNA channel) are calculated for each sub-array and the median value set to zero as our assumption is that the majority of genes are unchanging. As different gene microarray layouts may be employed, the requisite spatial information is retrieved from the microarray files themselves.

#### 5 - Average any multiple features

Where there are multiple copies of an identical sequence (feature) on a given microarray, the average of the log_2_-ratios is calculated so that any given feature appears just once in the dataset derived from that microarray.

#### 6 - Cyclic loess normalisation (optional)

Global loess normalisation is carried out on each pair of microarrays using *limma*. The 'span' parameter (the amount of data included for each local estimate) may also be user-defined; the default 'span' is 0.3, or 30%.

#### 7 - Average the normalised log_2_-ratios

The average normalised log2-ratio expression value for each feature across the dataset is calculated (see below). *BABAR *also handles the case where, inevitably, the fluorescent signal representing a given gene may not appear on every microarray (see below).

#### 8 - Correct medians to M = 0 (optional)

The averaged, normalised log_2_-ratios undergo a final correction such that the median log_2_-ratio of expression for each microarray is set to M = 0 (i.e. equal expression). Results may be outputted as a text file for the convenience of the user.

To better explain the steps following pre-processing of the data and especially the cyclic loess normalisation step, consider steps 6-8 in more detail with an example where *BABAR *is applied to a dataset comprised of three microarrays (*a*, *b *and *c*). For each feature on a microarray let *R *be the fluorescent signal channel and *G *the reference channel. A pair-wise MA-plot global loess normalisation is carried out on the first pair of microarrays (*a *vs. *b*) such that for each feature

The normalised log_2_-ratio (*N*_*ab*_) for each feature is calculated as *N*_*ab *_= *M*_*ab *_- loess (*A*_*ab*_), where loess(*A*_*ab*_) is the global loess curve. The reverse comparison (*b *vs. *a*) is simply given by *N*_*ba *_= - *N*_*ab*_.

The global loess step is repeated for the other pair-wise comparisons (*a *vs. *c*, *b *vs. *c*) followed by calculation of the reverse comparisons (*c *vs. *a*, *c *vs. *b*).

The normalised log_2_-ratios for each feature are averaged such that

where *n *is the number of pair-wise comparisons involving microarray *a *for which the feature was present. If the feature were present on all three microarrays, then *n *= 2 as two pair-wise comparisons can be made (*a *vs. *b*, *a *vs. *c*); the reverse comparisons are not counted (*b *vs. *a*, *c *vs. *a*). If the feature were only present on microarrays *a *and *b *then *n *= 1, as only one pair-wise comparison was made, and the normalised log-ratio for this feature on microarray *c *would be assigned NA.

The averaging of the normalised log_2_-ratios step is repeated for the other comparisons to obtain _*b *_(i.e. using *N*_*ba *_and *N*_*bc*_) and _*c *_(i.e. using *N*_*ca *_and *N*_*cb*_).

Finally, as it is our assumption that the majority of features are not differentially expressed, the averaged, normalised log_2_-ratios from each microarray undergo a final correction such that the median log_2_-ratio of expression for each microarray has an M- value of zero.

## Results and Discussion

*BABAR *was developed primarily to automate the normalisation of heterogeneous datasets (datasets containing different microarray file formats and gene array layouts). It handles 'problem' datasets (marked differences in the scale of M-values between microarrays perhaps due to issues with RNA quality) so they can be directly compared to 'good' datasets (M-value scaling is more comparable between microarrays due to good quality RNA derived from *in vitro*-grown organisms).

### Validating the *BABAR *algorithm

We first normalised a heterogeneous 'problem' dataset and compared our *BABAR*- normalised log_2_-ratios of gene expression to published RT-PCR experimental data. Normalising such a dataset with existing methods, if even possible, would have required substantial additional programming. Second, we applied *BABAR *to a non-heterogeneous 'good' dataset and compared the performance of the software in determining differentially expressed genes compared to a combination of *limma*-implemented 'within' and 'between' arrays normalisation. Finally, we applied *BABAR *to a modest, non-heterogeneous simulated dataset so as to compare its performance (power and type I error rates) with the *limma*-implemented methods.

### Assumptions

To achieve the simplification of the normalisation process via *BABAR *three key assumptions were made: that the majority of genes are not differentially expressed between experimental datasets (an assumption made by loess normalisation), that the same reference is employed throughout such that values in the gDNA channel always have a higher median than the signal channel (to allow dye-swaps to be handled) and that it is always appropriate to background-correct the microarray spots (i.e. genes). Regarding the latter assumption, our approach was to statistically filter the spots, background-subtract those that pass the filter (using these for our analyses) and discard those spots that fail. Low-intensity spots become more prone to variability as their measured signal values approach background. For our analyses we only used spots with intensities that were significantly different to the background; at 99.73% confidence, spots to be included in the analyses possessed intensities in excess of three standard deviations above the background, exceeding the recommended two standard deviations [20].

### Handling missing normalised data

The results file outputted from *BABAR *is in the form of a matrix of log_2 _normalised gene expression values for genes (rows) under a variety of experimental conditions (columns). It is inevitable that there will be missing values due to either experimental issues or the different gene array layouts employed between conditions. Missing value reconstruction is therefore necessary as many of the microarray analytical algorithms cannot handle missing values; they are typically replaced with zeros or with a row average. We used a better approach: the K-Nearest Neighbours Impute algorithm (KNNimpute) [21]. This considers the correlation structure of the data, finding and using those genes with the most similar expression profiles to the gene under investigation to impute any missing values.

### Putting a 'problem' dataset through *BABAR*

*BABAR *was applied to a heterogeneous 'problem' dataset from a study describing the transcriptomic changes for *Salmonella enterica *serovar Typhimurium internalised within mammalian host cells (epithelial cells or macrophages) as they adapted to the intracellular environment for different post-infection times [13] (ArrayExpress accession number E-MEXP-1368). This dataset was derived from RNA samples of varying quality, and generated from 23 common reference design GenePix-scanned microarrays described by a total of three gene microarray layouts. The unprocessed microarray data files are available as Additional file 3.

**Figure 1:**
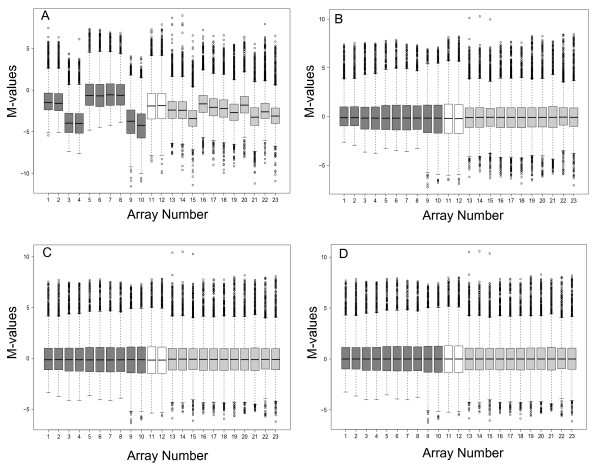
***BABAR*-generated boxplots showing *Salmonella *gene expression for the dataset of Hautefort *et al***. [13]. The figures plot M-value versus microarray identity. Gene expression values were obtained for *Salmonella *internalised in epithelial cells (dark grey boxplots), grown in Luria-Bertani broth (LB) control samples (white boxplots) or internalised in macrophages (light grey boxplots) prior to *BABAR *processing (A), and after background correction with 'within arrays' block-by-block data centering (B), cyclic loess with averaging of the log_2_-ratios (C), and final centering of the data representing the processed output of *BABAR *(D). Each box highlights the median and characterises the range of 50% of the data for each microarray; circles show individual genes that are outliers. Gene expression levels in epithelial cells were determined at 2 h (microarrays 1-4), 4 h (5-6) and 6 h (7-10) post infection (p.i.), for a mid-log LB culture (11-12), and in macrophages at 4 h (16-19), 8 h (20-23) and 12 h (13-15) p.i.

*BABAR *was run with the three normalisation steps enabled: block-by-block centering of the medians around M = 0, cyclic loess and a final correction of each microarray's median to M = 0. Four boxplots plotting M-value versus microarray ID were generated to represent the data prior to processing and after each of the three normalisation steps (Figure 1).

Prior to *BABAR *processing, the microarray data exhibited considerable variation around M = 0 (Figure 1A). Clearly, without normalising these data any gene expression comparisons made between microarrays would be meaningless.

The first steps implemented by *BABAR *in normalising the dataset were background correction and block-by-block data centering (median M-values corrected to zero) for all microarrays (Figure 1B). However, differences in the scale and distribution of M-values between arrays still remained, and were particularly pronounced between the macrophage (light grey boxplots; typical standard deviation, s.d. = 1.71) and control experiments (white boxplots; typical s.d. = 2.33). Another potential problem highlighted in Figure 1B concerned microarrays 1-8 (dark grey boxplots). Even though these data are log-transformed and pre-processed, they exhibit a (positively) skewed distribution nearly double that of the other microarrays (typical skewness, S_k _= 0.96 c.f. S_k _= 0.53). Clearly these data must be further transformed before robust comparisons between microarrays can be made.

After applying cyclic loess normalisation to MA-plots for all unique pairs of common reference microarrays, *BABAR *averages the log_2_-ratios (Figure 1C). As a result, the scale of the normalised M-values and the degree of skewness were made more comparable across the whole dataset; the macrophage (light grey boxplots) and control experiments (white boxplots) now had typical standard deviations of s.d. = 1.75 and s.d. = 2.04, respectively, and the typical skewness values for microarrays 1-8 and the remainder of the microarrays were S_k _= 0.87 and S_k _= 0.68, respectively (Figure 1C).

As it is assumed that the majority of genes are not differentially expressed (i.e. M = 0), *BABAR *carries out a final data centering (Figure 1D) as it may be seen that there has been a slight shift in the medians across the dataset away from M = 0 (highlighted by microarrays 12-13 in Figure 1C).

**Figure 2:**
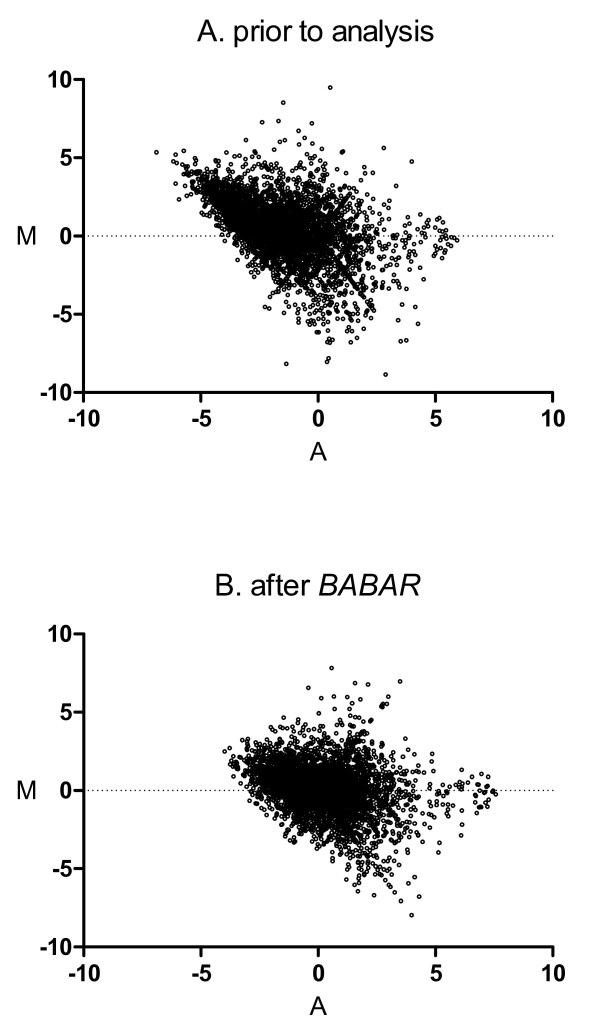
**MA-plots for a pair of microarrays before (A) and after (B) *BABAR***. For a pair of microarrays (array 1 vs. array 11 - see Figure 1) from [13], the intensity log-ratio M = log_2_{(R_1_/G_1_)/(R_11_/G_11_)} was plotted against the mean log intensity A = log_2 _√{(R_1_/G_1_) × (R_11_/G_11_)} for each gene.

Loess normalisation is a 'within' microarrays normalisation method that is used to normalise two channels on the *same *microarray (i.e. a direct comparison design). As described previously, we have conceptualised the comparison of a pair of common reference microarrays to be pseudo-direct; the loess algorithm is used here to normalise each unique pair of common reference microarrays. The MA-plots of Figure 2 are typical of those obtained from pairs of common reference two-colour microarrays. Figure 2A shows just one of the 253 (i.e. 23(23-1)/2) unique comparisons that *BABAR *made for this dataset, and Figure 2B shows that the overall effect of the *BABAR *algorithm on the same pair of microarrays is to improve the symmetry of the data around M = 0.

**Figure 3:**
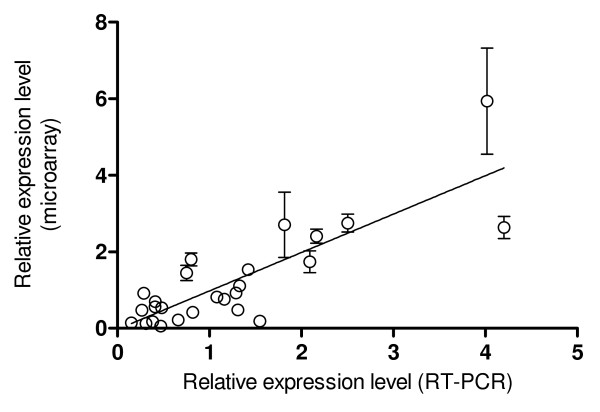
**Comparison of *BABAR*-normalised microarray data with RT-PCR analyses**. The relative expression levels for 13 *Salmonella *genes from inside epithelial cells (2 h p.i. or 6 h p.i. vs. mid-exponential LB control cultures) as determined by microarray analysis (*BABAR *normalisation) and RT-PCR are shown (Pearson correlation coefficient = 0.83, p < 0.001; r^2 ^= 0.69 via linear regression). For the RT-PCR analyses, RNA was independently extracted from LB cultures of *Salmonella *or from *Salmonella *cells internalised in epithelial cells, and used in RT-PCR amplification of the genes of interest.

The requirement for the microarrays in a dataset to have a similar spread and distribution prior to analysis is often seen as a 'must'. However, this is an assumption and the question of how best to normalise heterogeneous datasets without removing biological variation is an important one. It has been shown above that *BABAR*, by implementing the cyclic loess algorithm (a 'complete data method'), improves the symmetry of the MA-plot for microarray pairs and makes the distributional measures such as standard deviation and skewness closer over the dataset as a whole. Therefore validation was required to demonstrate that *BABAR*'s transformation of these data allowed for their correct interpretation. The *BABAR*-normalised results were outputted as a .txt file and the expression levels for 13 key genes were compared with the corresponding RT-PCR data from *Salmonella *cells internalized in epithelial cells at 2 h and 6 h post infection [13]. These genes, possessing a wide range of expression levels, are representative of those involved in iron uptake (*entB*), invasion (*invF*, *prgH*, *sifA*, *ssaG*), metabolism (*gapA*, *pgi*, *zwf*), the flagellar system (*flgL*, *fliC*, *fliF*, *fljB*), and included a control (*nusG*). It may be seen from Figure 3 that the microarray and RT-PCR data are in good agreement (Pearson correlation coefficient = 0.83, p < 0.001).

Additional validation was obtained at the level of protein expression. The results of immunogold and immuno-fluorescent labelling showed that the flagellin proteins FliC and FljB were indeed present in infected epithelial cells at detectable levels [13], and a two-fold increase in FliC protein was observed inside epithelial cells (6 h c.f. 2 h) which compared to ~three-fold increase for the microarray (3.3-fold) and RT-PCR data (3.6-fold). These findings validated the *BABAR*-normalisation of the original dataset because the protein data supported the transcriptomic findings.

Though modest in scale, this dataset presented a real challenge to other existing software because the constituent microarrays were described by three gene array layouts and showed considerable M-value variation. We are unaware of any software that automatically handles such data. Our findings validate the *BABAR *algorithm for the normalisation of heterogeneous datasets prior to further analyses.

### *BABAR *vs. standard techniques

**Figure 4:**
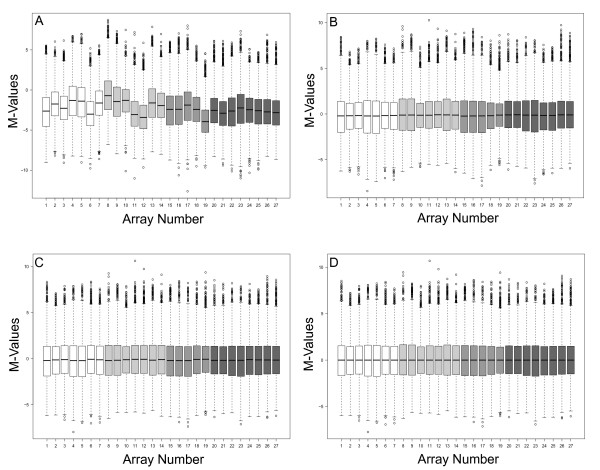
***BABAR*-generated boxplots showing *Salmonella *gene expression for the IHF dataset **[22]. The figures plot M-value versus microarray identity. Gene expression values were obtained for wild-type *Salmonella *(white boxplots), IHFα mutant (light grey boxplots), IHFβ mutant (grey boxplots) and the double IHF mutant (dark grey boxplots) prior to *BABAR *processing (A), after background correction with 'within arrays' block-by-block data centering (B), cyclic loess with averaging of the log_2_-ratios (C), and final centering of the data representing the processed output of *BABAR *(D). Each box highlights the median and characterises the range of 50% of the data for each microarray; circles show individual genes that are outliers.

We next compared the performance of *BABAR *in determining differentially expressed genes with standard methods as implemented by another R package, *limma *(version 2.16.4). We applied both packages to a dataset describing the transcriptomic changes for virulence-related genes at different phases of growth for the wild-type (WT) *S*. Typhimurium compared to mutants deficient in one or both subunits of the DNA- binding protein Integration Host Factor (IHF), IHFα and IHFβ [22] (ArrayExpress accession number E-MEXP-2416). As the bacteria were grown *in vitro*, RNA quality for this dataset was good. This dataset was generated from 27 common reference design GenePix-scanned microarrays described by a single gene array layout (see Additional file 4 for the unprocessed microarray data files and the associated .gal file). *BABAR *was run with the main normalising steps enabled and generated the boxplots seen in Figure 4.

**Figure 5:**
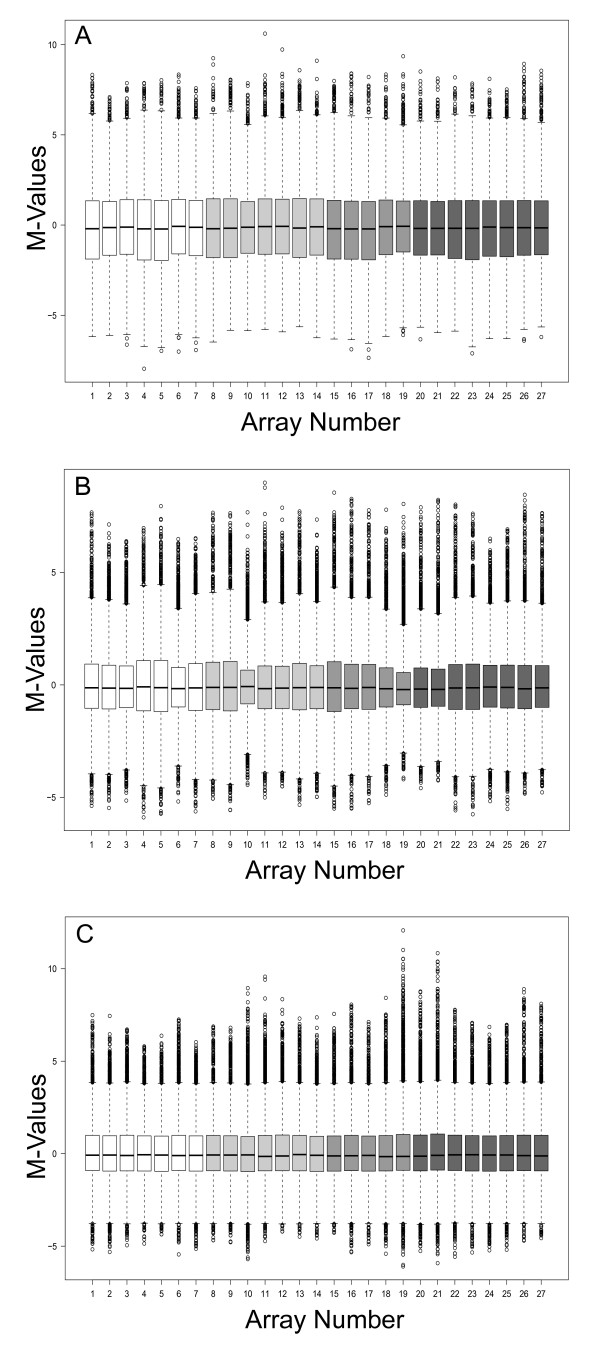
**Comparing the spread of expression values across the IHF dataset **[22]**after *BABAR *and *limma*-normalisation**. The figures plot M-values versus microarray identity (see Figure 4 legend for details). Gene expression values were obtained after processing with *BABAR *(with no final centering; A), *limma *quantile normalisation (B) and *limma *scale normalisation (C). Each box highlights the median and characterises the range of 50% of the data for each microarray; circles show individual genes that are outliers.

Although still requiring normalising, the IHF dataset is an example of a 'good' non-heterogeneous dataset that was derived from RNA that was of a consistently good quality. Compared to Fig 1A, there is a reduction in the variation about M = 0, skewness (typical S_K _= 0.32) and variance of the microarrays M-values (Figure 4A). Following *BABAR*'s three normalisation steps (Figures 4B, 4C and 4D) these data were ready for analysis. For *limma *normalisation we applied background correction (normexp) and a 'within arrays' method for data centering (printtiploess) followed by a 'between arrays' method, either quantile or scale normalisation. The slight median shift away from M = 0 that was apparent after *BABAR*'s cyclic normalisation step (Figures 4C and 5A) was also seen following quantile (Figure 5B) or scale normalisation (Figure 5C). This was not 'corrected' for in the latter two methods as an equivalent step is not implemented by the *limma *package.

The published IHF study focused primarily on the comparison between the WT and the *ihfAB *double mutant, with one of the key findings being that the loss of IHF had strong negative effects on virulence genes [22]. Our focus here will therefore be on down-regulation of gene expression. To determine the differentially expressed down- regulated genes for *BABAR*- and *limma*-normalised log_2_-ratios, the outputs from these methods were analysed via the Qlucore Gene Expression Explorer (QGEE) software (version 1.1; [3]). The QGEE software was used to impute any missing values via its implementation of the KNNimpute algorithm (default number of neighbours used in the imputation = 10) [21] and to identify those down-regulated genes that could separate the WT and double mutant samples with a high degree of confidence (p ≤ 0.01) for each method.

**Figure 6:**
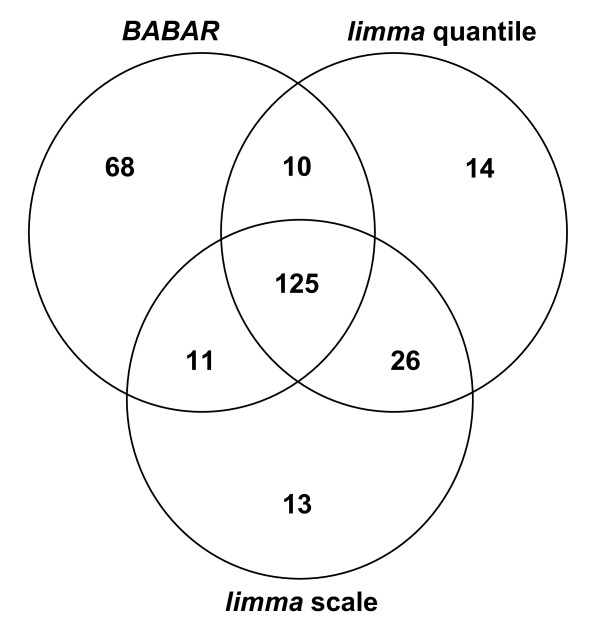
**A comparison of down-regulated genes detected after *BABAR*, scale and quantile normalisation**. A Venn diagram showing the relations between down-regulated genes detected by the QGEE software (p ≤ 0.01) for the IHF dataset [22] (WT versus *ihfAB *double mutant) following either *BABAR*, *limma *scale or *limma *quantile normalisation. For the *BABAR *processing, the QGEE software analyses were run after background correction with 'within arrays' block-by-block centering of the medians, 'between arrays' cyclic loess with averaging of the log_2_-ratios, and final centering of the data. For the two *limma*-implemented normalisation methods the QGEE software analyses were run after the data were background corrected (normexp) with a 'within arrays' method for data centering (printtiploess), followed by a 'between arrays' method, either quantile or scale normalisation.

Interestingly, despite the suggestion from the boxplots (Figures 5B and 5C), the results from the QGEE software analyses after scale or quantile normalisation were very similar. From Figure 6 it may be seen that at p ≤ 0.01 both methods detected 175 down-regulated genes with 151 genes (~86 %) common to both. Comparably, *BABAR *detected 214 down-regulated genes with 136 and 135 genes in common with scale and quantile normalisation, respectively. For the genes that were the focus for the original IHF study (see below), the quantile normalisation method detected two genes that were absent from the scale normalisation results (results not shown). Therefore, for simplicity only the quantile data was considered further and will be referred to as the *limma *data.

Our comparison of normalisation methods focused on differences in expression between the WT and double *ihfAB *mutant for genes encoded by the four *Salmonella *Pathogenicity Islands (SPI1, 2, 4 & 5), chemotaxis and flagellae genes. A summary of the results for the differential gene expression analysis of virulence-associated genes shows excellent agreement between *BABAR *and *limma *(Table 1). For example, for the 40 genes encoded by the SPI1 pathogenicity island, both *BABAR *and *limma *determined 29 genes to be down-regulated, with 28 of those genes common to both. Closer inspection showed the QGEE software analysis detected slightly more down-regulated genes for the *BABAR*-normalised data (64 genes) compared to the *limma*-normalised data (59 genes).

**Table 1:** Validation of *BABAR *by comparison with *limma *normalisation for the detection of differentially-expressed genes

	Number of down-regulated genes detected		
	
Virulence genes	*BABAR *unique	Both methods	*limma *unique
SPI1 (40)	1	28	1
SPI2 (31)	4	10	2
SPI4 (6)	1	4	1
SPI5 (11)	1	3	0
**Other genes**			
			
Chemotaxis (9)	1	5	0
Flagellae (23)	2	4	1

The number of down-regulated genes detected by the QGEE software (p ≤ 0.01) for the IHF dataset [22] (WT versus *ihfAB *double mutant) following either *BABAR *or *limma *quantile normalisation. The number of genes in each group is given in brackets (120 in total). Chemotaxis genes include *che *and *mot *genes; flagellae genes include *flg*, *fli *and *flj *genes.

These results show that the *BABAR *algorithm can be successfully used to normalise datasets prior to further analyses.

### Comparison of methods using simulated data

To further validate *BABAR *we compared its performance against the *limma*-implemented methods for normalisation using a simulated dataset. With such datasets, the number of differentially expressed genes is known making it possible to calculate the power of detection and type I errors. *SIMAGE *was used to simulate two-colour DNA microarray data [23], generating 6 simulated microarrays each with 5000 genes arranged in 4 × 4 blocks; *SIMAGE *default settings were otherwise used. Due to the difficulty described previously in analysing heterogeneous datasets these simulated data were treated as consisting of single format result files described by a single array layout. The output files were converted to a .gpr format and normalised via *BABAR*, print-tip loess, quantile and scale normalisation. Significance analysis of microarrays (SAM [24]) was carried out using the freely accessible *samr *R package (version 1.26) [25] to calculate power (1 - false discovery rate) and type I error (false negative) rates for each normalisation method.

It may be seen from Table 2, that for 118 differentially expressed genes the performance of *BABAR *(power = 0.62) was comparable to scale normalisation (power = 0.66), and was considerably better than quantile (power = 0.51) or print-tip loess (power = 0.48) normalisation. At higher numbers of differentially expressed genes, scale normalisation appeared to perform best but *BABAR *continued to outperform both quantile and print-tip loess normalisation. It was also seen that the three 'between arrays' methods outperformed the print-tip loess 'within arrays' method. One issue with simulated datasets is that they can't inform upon what level of power is required to still obtain the correct biological interpretation for a given dataset; perhaps detecting only the 10% most regulated genes, a power as low as 0.1, may be sufficient for some datasets [26]. When comparing normalisation methods it is important to analyse real and simulated data, and our findings have shown *BABAR*'s effectiveness in highlighting differentially expressed genes from real datasets. The performance of *BABAR *is impressive given that it was designed with large, heterogeneous datasets in mind (not the modest and uniformly designed simulated dataset described above) and makes limited use of any spatial information.

**Table 2:** Comparison of the power and type I errors for a simulated microarray dataset

	Normalisation method							
	
	Scale		*BABAR*		Quantile		print-tip loess	
**No. of genes**	**Power**	**Error**	**Power**	**Error**	**Power**	**Error**	**Power**	**Error**

118	0.66	0.022	0.62	0.030	0.51	0.032	0.48	0.033
220	0.73	0.034	0.58	0.053	0.57	0.051	0.51	0.056
407	0.77	0.055	0.62	0.088	0.61	0.089	0.56	0.091
756	0.82	0.088	0.67	0.150	0.65	0.158	0.63	0.163
1402	0.87	0.144	0.76	0.260	0.74	0.300	0.71	0.314

The power and proportion of type I errors for a dataset consisting of 6 simulated microarrays [23] were calculated via the *samr *R package [25]. Analysis followed one of four methods of microarray data normalisation for different numbers of differentially expressed genes. For *BABAR*, analyses were run after background correction (subtraction) with 'within arrays' block-by-block centering of the medians, 'between arrays' cyclic loess with averaging of the log_2_-ratios, and final median centering of the data. For the other methods, analyses were run after the data were background corrected (normexp) with a 'within arrays' method for data centering (printtiploess), followed by the appropriate 'between arrays' method (none, quantile or scale normalisation).

### Normalisation comparison summary

We have demonstrated *BABAR*'s ability to transform microarray data compares well with the standard methods investigated so as to allow for their correct interpretation. As *BABAR *implements an algorithm which normalises each unique pair of microarrays, the impact on the running time of the analysis was investigated. For a PC running Windows XP with a 2.66 GHz Intel^®^Core™ 2 Duo CPU with 2 GB RAM, *BABAR *analysis of the IHF dataset (27 microarrays) took 40s compared to 15s via the *limma*-implemented methods. Cyclic loess is still preferred to quantile normalisation, for example, as it is not as aggressive in its normalisation, but if run time issues are a concern then a faster cyclic loess technique has been developed [16].

In using real and simulated datasets for a direct comparison of *BABAR *with *limma*-implemented methods of normalisation, it has been necessary to use non-heterogeneous datasets. This is due to the fact that the latter methods are unable to handle the heterogeneous datasets for which *BABAR *was developed. To handle such datasets *BABAR *makes some assumptions about the data it will receive, but *BABAR's *real value comes from handling those datasets that other applications cannot.

### Present and future applications of *BABAR*

Following *BABAR*-normalisation, it is possible to compare the data derived from any microarray with any other for a given dataset with confidence. Other studies have employed very different approaches to the challenge of combining different microarray data into a single dataset which may then be subjected to further analysis. One study used multivariate linear regression to create a fused and cleaned dataset from data for 9 experiments (178 microarrays), downweighting microarrays of poor experimental quality [27]. Another study used statistical discrimination to merge two datasets from different microarray platforms (cDNA and Agilent oligo microarrays) [28]. However, both of these methods require the microarrays to have been normalised prior to merging. In contrast, a further study [29] employed a novel three-step strategy to normalise *and *integrate data arising from five Affymetrix microarray generations. Using a different strategy *BABAR *also normalises and merges microarray data but is applied to common reference two-colour microarrays with different file formats or array layouts.

Our focus in this paper has been on differential gene expression, which is important for many experimentalists. We believe that great benefit will be also be obtained when applying *BABAR *to datasets prior to 'systems'-level analyses which, for instance, utilise large transcriptomic datasets to infer regulatory or metabolic networks. Like many research groups we have amassed a vast collection of transcriptomic data into a gene expression compendium and network inference techniques are now being used to extract high-level information from these data [10,30]. However, the initial normalisation of these data is critical if meaningful interpretations are to be made.

We are not aware of any software that would handle the heterogeneous datasets that can comprise such gene expression compendia without considerable additional programming. Our recent network analysis of a compendium of heterogeneous two-colour transcriptomic data demonstrated the value of the *BABAR *algorithm. At present *BABAR *accepts only (unprocessed) GenePix and BlueFuse microarray data files; a useful adaptation would allow other data file formats to be processed. In addition, enabling *BABAR *to handle tables of (un-normalised) data, as might be retrieved from repositories such as ArrayExpress or the Gene Expression Omnibus, would greatly extend the reservoir of data for analysis.

## Conclusions

The development of DNA microarrays for transcriptomic analysis has revolutionised many areas of biology and has facilitated the generation of a mass of transcriptomic datasets. The normalisation of such datasets remains a challenge as many factors can contribute to the variability that occurs between microarrays, confounding the biologically-correct interpretation of these data. The challenge is compounded by the heterogeneous datasets that may be compiled for 'systems'-level analyses. The surge in interest in deep-sequencing techniques (e.g. [31] for a recent review of the area applied to microbial genetics) may seem to have heralded the 'death of microarrays' [32]. However, given the amount of transcriptomic data that await analysis, microarrays will remain to be a powerful analytical technique for some time. As this is an ideal tool for facilitating robust gene expression and network inference analyses, to obtain maximum insight from valuable transcriptomic data use *BABAR*.

## Availability and requirements

Project name: *BABAR*

Project home page: http://www.ifr.ac.uk/info/science/FoodbornePathogens/index.htm

Operating System(s): Windows XP and Red Hat Enterprise Linux 5.

Programming language: R

Other requirements: R-2.9.2 or newer, and *limma *2.16.4 or newer.

License: GNU General Public License

*BABAR *is available as a zip file (see Additional file 1) with detailed installation and running instructions (see Additional file 2).

## Authors' contributions

JS wrote the source code and created the package. MJA tested the package, analysed and interpreted the data, drafted the manuscript and assisted JS. SL supplied the test data and with JCDH was involved in the conception of this research and finalising the manuscript. All authors read and approved the final version of the manuscript.

## Supplementary Material

Additional file 1 ***BABAR *software**. The *BABAR *R package. Click here for file

Additional file 2 ***BABAR *documentation**. Vignette detailing the installation and running of *BABAR*. Click here for file

Additional file 3 **Dataset 1**. The unprocessed microarray data files from a heterogeneous 'problem' dataset describing the transcriptomic changes for *Salmonella *internalised within mammalian cells. Click here for file

Additional file 4 **Dataset 2**. The unprocessed microarray data files and the associated .gal file for a non-heterogeneous 'good' dataset describing the transcriptomic changes of virulence genes for wild-type *Salmonella *and IHF mutants. Click here for file
