# Supplementary material for: BABAR: an R package to simplify the normalisation of common reference design microarray-based transcriptomic datasets
Source: BMC Bioinformatics. 2010 Feb 3;11:73. doi: 10.1186/1471-2105-11-73 (PMC2829013; doi:10.1186/1471-2105-11-73)
Supplement: Additional file 1 — BABAR software. The BABAR R package. [file 1471-2105-11-73-S1.ZIP › babar/html/00Index.html]

R: Multiple gal files, bluefuse and genepix

# Multiple gal files, bluefuse and genepix

---

## Documentation for package ‘babar’ version 1.7

## Help Pages

|  |  |
| --- | --- |
| babar-package | Multiple gal files: bluefuse and genepix |
| allnorm | Internal routines of babar - not for general use |
| avexpr | Internal routines of babar - not for general use |
| babar | Combining Bluefuse and Genepix microarrays |
| babar-internal | Internal routines of babar - not for general use |
| BABARVERSION | Combining Bluefuse and Genepix microarrays |
| bluetopix | Internal routines of babar - not for general use |
| BOXPLOTS | Combining Bluefuse and Genepix microarrays |
| doblue | Internal routines of babar - not for general use |
| doblue | Internal routines of babar - not for general use |
| doboxplotsA | Internal routines of babar - not for general use |
| doboxplotsB | Internal routines of babar - not for general use |
| doboxplotsB | Internal routines of babar - not for general use |
| dopix | Internal routines of babar - not for general use |
| FINALCENTRE | Combining Bluefuse and Genepix microarrays |
| finalcentre | Internal routines of babar - not for general use |
| LOESS | Combining Bluefuse and Genepix microarrays |
| matchexpr | Internal routines of babar - not for general use |
| multgal | Internal routines of babar - not for general use |
| multgalcomb | Internal routines of babar - not for general use |
| multgalloess | Internal routines of babar - not for general use |
| multgalunique | Internal routines of babar - not for general use |
| preprocess | Internal routines of babar - not for general use |
| RUNSTAMP | Combining Bluefuse and Genepix microarrays |
| sachamediannorm | Internal routines of babar - not for general use |
| sachanormfile | Internal routines of babar - not for general use |
| sachanormfiles | Internal routines of babar - not for general use |
| SPAN | Combining Bluefuse and Genepix microarrays |
| uniquegenes | Internal routines of babar - not for general use |
