# Supplementary material for: BABAR: an R package to simplify the normalisation of common reference design microarray-based transcriptomic datasets
Source: BMC Bioinformatics. 2010 Feb 3;11:73. doi: 10.1186/1471-2105-11-73 (PMC2829013; doi:10.1186/1471-2105-11-73)
Supplement: Additional file 1 — BABAR software. The BABAR R package. [file 1471-2105-11-73-S1.ZIP › babar/html/babar-internal.html]

R: Internal routines of babar - not for general use

|  |  |
| --- | --- |
| babar-internal {babar} | R Documentation |

## Internal routines of babar - not for general use

### Description

Takes genepix and/or bluefuse microarray files, matches up
the genes and normalise.

### Details

|  |  |
| --- | --- |
| Package: | babar |
| Version: | 1.0 |
| Date: | 28/02/2008 |
| Depends: | R, utils, limma, chron, RDCOMClient |
| Suggests: |  |
| LazyLoad: | no |
| License: |  |
| URL: |  |
| Packaged: | Wed Feb 20 15:06:24 2008; seersj |
| Built: | R 2.6.2; ; 2008-02-20 15:08:43; windows |

### Author(s)

John Seers John.Seers@bbsrc.ac.uk
Maintainer: John Seers <John.Seers@bbsrc.ac.uk>

### References

http://www.ifr.ac.uk

### Examples

```
## Not run: 
## Run a normalisation on array files
# Set the base dir
basedir<-"C:/temp"
# Set the working directory to be used
setwd(basedir)
# Files are tab delimited. (Not Excel).
# Set the list of bluefuse files to be processed
bluefiles<-dir(basedir, pattern=".xls$", full.names=TRUE)
# Set the list of genepix files to be processed
genepixfiles<-dir(basedir, pattern=".gpr$", full.names=TRUE)

# Run the multiple gal file ratio of ratios processing
ratiodata<-babar(bluefiles, genepixfiles)
## End(Not run)
```

---

[Package *babar* version 1.7 Index]
