# Supplementary material for: BABAR: an R package to simplify the normalisation of common reference design microarray-based transcriptomic datasets
Source: BMC Bioinformatics. 2010 Feb 3;11:73. doi: 10.1186/1471-2105-11-73 (PMC2829013; doi:10.1186/1471-2105-11-73)
Supplement: Additional file 1 — BABAR software. The BABAR R package. [file 1471-2105-11-73-S1.ZIP › babar/html/babar.html]

R: Combining Bluefuse and Genepix microarrays

|  |  |
| --- | --- |
| babar {babar} | R Documentation |

## Combining Bluefuse and Genepix microarrays

### Description

Combining arrays and normalising

### Usage

```
babar(bluefusefiles, genepixfiles)
```

### Arguments

|  |  |
| --- | --- |
| `bluefusefiles` | a list of bluefuse files |
| `genepixfiles` | a list of genepix files |

### Details

There are global variables that can be set or viewed:  
BABARVERSION - version number of babar  
LOESS - TRUE/FALSE - default TRUE - perform a loess multgal operation  
FINALCENTRE - TRUE/FALSE - default TRUE - perform a final median centering after the loess  
RUNSTAMP - file time stamp for a unique name  
SPAN - span value used for loess - default 0.3 to match limma loessFit  
BOXPLOTS - TRUE/FALSE - default FALSE - produce boxplots of expression values  
NUMBEROFSD - n - default 3 - specify the number of standard deviations away to flag data  
REFERENCEDETECTION - default "OFF" - uses array headers to determine genomic DMNA column. Else does it automatically.

### Value

A list of expression values, gene names

### Author(s)

John Seers John.Seers@bbsrc.ac.uk

### References

http://www.ifr.ac.uk

### Examples

```
## Not run: 
## Run a normalisation on array files
# Set the base dir
basedir<-"C:/temp"
# Set the working directory to be used
setwd(basedir)

# Files are tab delimited. (Not Excel format).
# Set the list of bluefuse files to be processed
bluefusefiles<-dir(basedir, pattern=".xls$", full.names=TRUE)
# Set the list of genepix files to be processed
genepixfiles<-dir(basedir, pattern=".gpr$", full.names=TRUE)

# Run the multiple gal file ratio of ratios processing
ratiodata<-babar(bluefusefiles, genepixfiles)
## End(Not run)
```

---

[Package *babar* version 1.7 Index]
