# Supplementary material for: BABAR: an R package to simplify the normalisation of common reference design microarray-based transcriptomic datasets
Source: BMC Bioinformatics. 2010 Feb 3;11:73. doi: 10.1186/1471-2105-11-73 (PMC2829013; doi:10.1186/1471-2105-11-73)
Supplement: Additional file 2 — BABAR documentation. Vignette detailing the installation and running of BABAR. [file 1471-2105-11-73-S2.PDF]

BABAR: an R package to simplify the normalisation of common reference design microarray-based transcriptomic datasets

Mark Alston & John Seers

October 2009

Contents

Introduction 1

Preliminaries 2

Workflow 2

- Open R and load the required packages 2
- Set the base and working directories 2
- Select the required parameters 2
- Select the required normalisation steps 3
- Visualisation 3
- Set the type(s) of array files to be processed 3
- Run the *BABAR* normalisation and output the results . . 4

Example application 4

References 8

Introduction

The *BABAR* package (**B**atch **A**nti-**B**anana **A**lgorithm in **R**) automates the loading and normalisation of two-colour, common reference design microarrays (Alston *et al.*, 2009). *BABAR* accepts any combination of (unprocessed) GenePix and BlueFuse files, carrying out 'within' and 'between' array normalisation steps to make the arrays in a dataset comparable. *BABAR* returns boxplots (as .pdf files) for each of the normalisation steps and a data frame of the normalised $\log_2$ expression ratios which the user may save as a text file.

Preliminaries

BABAR has been tested on Windows XP and Red Hat Enterprise Linux 5. It requires you to have installed R version 2.9.2 (or later) and the Bioconductor package *limma* (http://www.bioconductor.org). To install *limma*, source biocLite from Bioconductor

```
> source("http://www.bioconductor.org/biocLite.R")
> biocLite("limma")
```

BABAR is supplied as a .zip file.
To install *BABAR* under Windows (e.g. RGui) select 'packages' and 'install package(s) from local zip file' from the menu. On Linux, unzip the file and move the resulting *babar* directory to the R library (/your/path/to…/R/library).

Finally, create a base directory (e.g. called `arrayFiles`) and unzip the example dataset (e.g. dataset1.zip) such that the array files to be normalised are in the base directory (see 'Set the base and working directories' below).

Workflow

Open R and load the required packages

```
> library(babar)
> library(limma)
```

Set the base and working directories

This allows R to locate the array files for normalisation in the base directory `basedir`.

```
> basedir<-"C:/babar/someExperiment/arrayFiles"
> setwd(basedir)
```

Select the required parameters

BABAR provides an option to automatically detect the reference channel.

```
> REFERENCEDETECTION="ON"     (otherwise set to "OFF")
```

For background correction, the number of standard deviations between a feature's background and foreground signals to pass the 'filter' may be set.

```
> NUMBEROFSD<-2               (default is 3SD)
```

For the cyclic loess, the span, or amount of data included for each local estimate, may also be set by the user.

```
> SPAN<-0.5                   (default is 0.3, or 30%)
```

Select the required normalisation steps

BABAR normalisation implements three main steps, making use of *limma*'s loess function (Smyth and Speed, 2003). The major assumption throughout is that the majority of genes will be unchanging in their level of expression.

Use these R queries

```
> LOESS<-TRUE
> FINALCENTRE<-TRUE
```

to implement all three normalisation steps:

(1) for a given array, each block is adjusted such that its median is zero (i.e. 'within' arrays normalisation),
(2) for each pair of arrays, a cyclic loess step is employed (i.e. 'between' arrays normalisation), and finally
(3) the median for each array is zeroed.

We would recommend running the options given above but the user is free to decide which they want to implement. If `LOESS<-FALSE` only (1) is run. Similarly, if `FINALCENTRE<-FALSE` only (1) and/or (2) are run.

Visualisation

To obtain diagnostic boxplots of the data, add the query:

```
> BOXPLOTS<-TRUE
```

This generates four boxplots, one for the unnormalised data and one for each of the three normalisation steps outlined above.
They are saved in the `basedir` as .pdf files. The boxplots for data prior to processing and after step (1) are saved under boxplot 'A', data after step (2) are saved as boxplot 'B' and data after step (3) are saved as boxplot 'C'.

Set the type(s) of array files to be processed

BABAR handles GenePix (.gpr) and BlueFuse (.xls) files. For a dataset containing both types of file:

```
> bluefiles<-dir(basedir, pattern=".xls$", full.names=TRUE)
> genepixfiles<-dir(basedir, pattern=".gpr$", full.names=TRUE)
```

Otherwise, replace the line with:

```
> bluefiles<-NULL
```
or
```
> genepixfiles<-NULL
```

Run the *BABAR* normalisation and output the results

The `babar` function runs the normalisation:

```
> ratiodata<-babar(bluefiles, genepixfiles)
```

The results are collated as a dataFrame of the normalised $\log_2$ ratios and may be saved as a (.txt) file in the user-defined destination directory `file`:

```
> results<-cbind(ratiodata[["genes"]], ratiodata[["exprs"]])

>  write.table(results,  file="C:/babar/someExperiment/babarOutput.txt",
+ sep="\t", col.names=NA)
```

As outlined above, a number of boxplots may be generated by *BABAR* and saved in the `basedir` as pdf files. To identify the arrays in the boxplots, run the following query to list the array names from `ratiodata`:

```
> colnames(ratiodata[["exprs"]])
```

Example

For the convenience of the user, here we reproduce an example for an R script to apply *BABAR* to a 'noisy' dataset (available as dataset1.zip). We assume the steps outlined under 'Preliminaries' have been implemented.
These data come from a study describing the transcriptomic changes for *Salmonella* cells internalised within host cells, either macrophages or epithelial cells (Hautefort *et al*., 2008; ArrayExpress accession number E-MEXP-1368). The 23 arrays of the dataset are common reference design, GenePix-scanned arrays and comprising of three gene array layouts.

```
# load the libraries
library(babar)
library(limma)

# Set the base dir i.e. where the array files are held
basedir<-"/somePath/babar/arrayData”

# Set the working directory to be used
setwd(basedir)

REFERENCEDETECTION="ON"
```

```
NUMBEROFSD<-3      ### default is 3
SPAN<-0.3          ### default is 0.3
LOESS<-TRUE
FINALCENTRE<-TRUE
BOXPLOTS=TRUE

# Set the list of bluefuse files to be processed, else set to NULL
#bluefiles<-dir(basedir, pattern=".xls$", full.names=TRUE)
bluefiles<-NULL

# Set the list of genepix files to be processed, else set to NULL
genepixfiles<-dir(basedir, pattern=".gpr$", full.names=TRUE)
#genepixfiles<-NULL

# Run the multiple gal file ratio of ratios processing
ratiodata<-babar(bluefiles, genepixfiles)
results<-cbind(ratiodata[["genes"]], ratiodata[["exprs"]])
write.table(results, file="/somePath/babar/output.txt”, sep="\t",
col.names=NA)

colnames(ratiodata[["exprs"]])
```

The R script runs all three *BABAR* normalisation steps, generating four boxplots (Figures 1-4) plotting M-value versus array ID. Each box highlights the median and characterises the range of 50% of the data for each array; circles show genes that are outliers.

Figure 1 Prior to processing

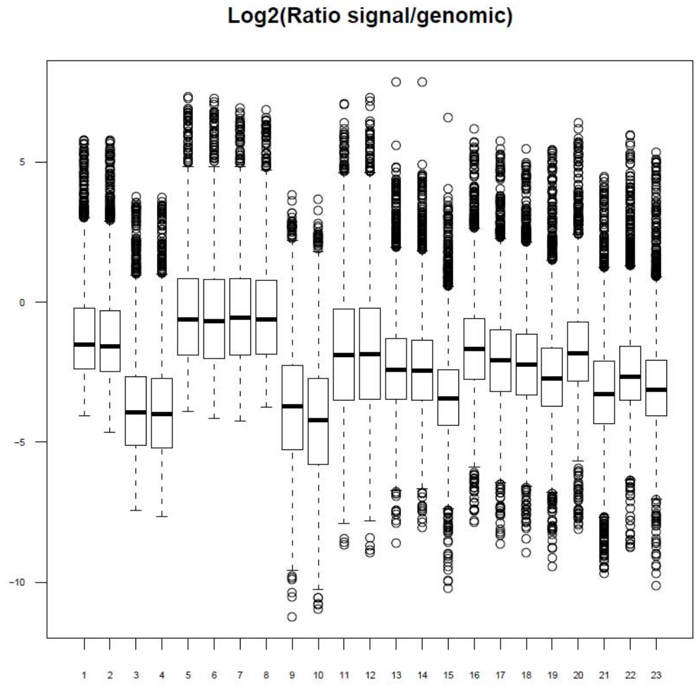

Figure 2 After adjusting the medians for each block within each array to zero

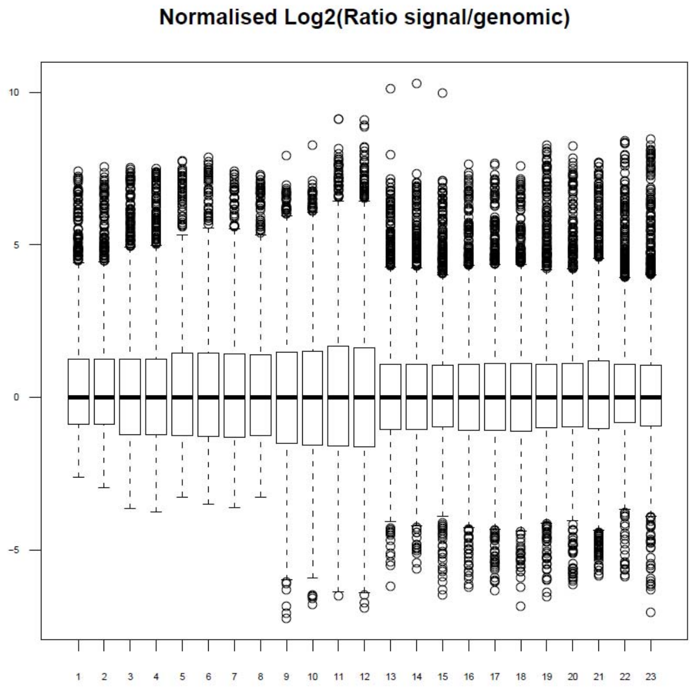

Figure 3 After cyclic loess normalisation and averaging

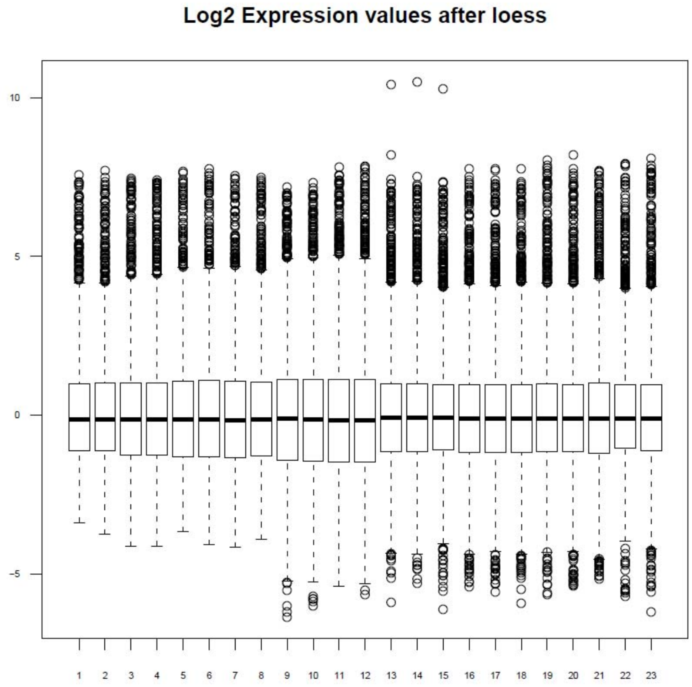

Figure 4 After adjusting the median for each array to zero

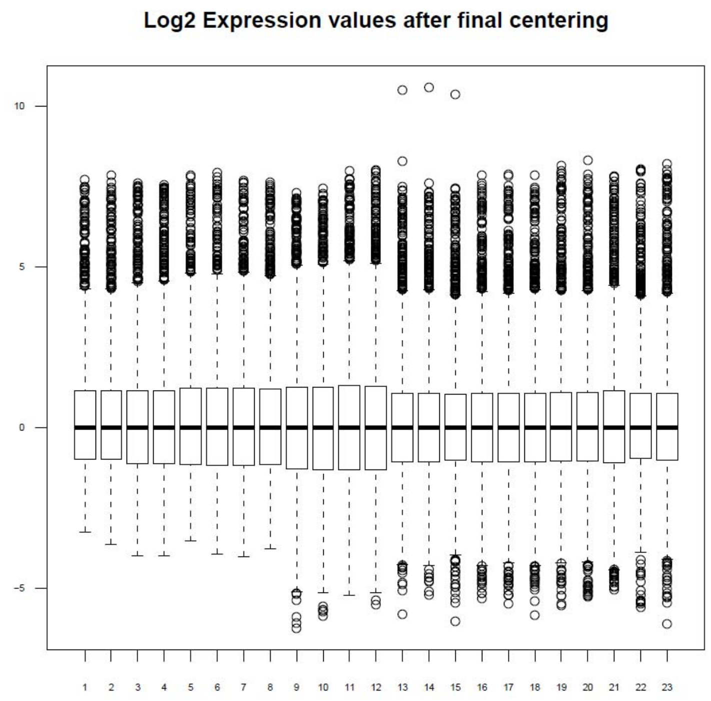

The boxplots are saved in the user-defined `basedir` as pdf files. To aid identification of the arrays in the boxplots, the last line of the script outputs the file name for each of the arrays. Note that arrays are outputted in alphabetical order on the boxplots, so it is recommended that microarrays are given ‘meaningful’ names.

```
> colnames(ratiodata[["exprs"]])
[1] "E2h-09A"  "E2h-09B"  "E2h-86A"  "E2h-86B"  "E4h-10A"  "E4h-10B"  "E6h-08A"  "E6h-08B"  "E6h-73A"
[10] "E6h-73B"  "LB_76A"   "LB_76B"   "M12h-75B" "M12h-79A" "M12h-80B" "M4h-75A"  "M4h-77B"  "M4h-80A"
[19] "M4h-91A"  "M8h-77A"  "M8h-92A"  "M8h-93B"  "M8h-98B"
```

In this example, the array file name prefix refers to epithelial cells (E), macrophages (M) or the Luria broth (LB) control. The other portion of the array file name refers to the incubation time and (part) of the slide number.

As RNA had to be isolated from internalised bacteria, in our experience the arrays would be expected to show considerable variation around M = 0 even after ‘within’ array normalisation (Fig. 2). After the cyclic loess normalisation step (Fig. 3) a very slight ‘drifting’ of the median values away from M = 0 can be seen (compare arrays 12 and 13). Therefore a final adjustment of each array is employed whereupon the *BABAR* normalisation can be seen to have made the spread of the M-values comparable across the whole data set (Fig. 4). The normalised $\log_2$ ratios may be saved as a (.txt) file in the user-defined destination directory `file`.

The normalised data obtained via *BABAR* for this dataset were validated via RT-PCR (see Alston *et al*., 2009).

References

Alston, M. *et al*. (2009) BABAR: an R package to simplify the normalisation of common reference design microarray-based transcriptomic datasets. *BMC Bioinformatics* (submitted)

Hautefort, I. *et al*. (2008) During infection of epithelial cells Salmonella enterica serovar Typhimurium undergoes a time-dependent transcriptional adaptation that results in simultaneous expression of three type 3 secretion systems. *Cell. Microbiol*. **10**(4), 958-984.

R Development Core Team (2008). R: A language and environment for statistical computing. R Foundation for Statistical Computing, Vienna, Austria. ISBN 3-900051-07-0, URL http://www.R-project.org.

Smyth, G. K., and Speed, T. P. (2003) Normalization of cDNA microarray data. *Methods* **31**, 265- 273.
